# Optimising the Orthopaedic Trauma Society Open Fracture Classification system: a proposal for modification in the context of high civilian gunshot fractures

**DOI:** 10.1007/s00590-024-03853-6

**Published:** 2024-02-22

**Authors:** Zamalunga Lunga, Maritz Laubscher, Simon Matthew Graham, Michael Held, Nando Ferreira, Ramanare Magampa, Sithombo Maqungo

**Affiliations:** 1 Orthopaedic Research Unit, Division of Orthopaedic Surgery, Groote Schuur Hospital, University of Cape Town, H49 Old Main Building, Observatory, Cape Town, 7925 South Africa; 2 Oxford Trauma and Emergency Care. Nuffield Department of Orthopaedics, Rheumatology & Musculoskeletal Sciences, University of Oxford, Oxford, UK; 3 Liverpool Orthopaedic and Trauma Service, Liverpool University Teaching Hospital Trust, Liverpool, UK; 4 Division of Orthopaedic Surgery, Groote Schuur Hospital, Cape Town, South Africa; 5 Division of Orthopaedic Surgery, Department of Surgical Sciences, Faculty of Medicine and Health Sciences, Stellenbosch University, Stellenbosch, South Africa; 6 Division of Global Surgery, University of Cape Town, Cape Town, South Africa

**Keywords:** Classification system, Femur, Gunshot fractures, Open fractures, Tibia

## Abstract

**Objectives:**

Uniformly classifying long bone open fractures is challenging. The purpose of this study was to propose a modified Orthopaedic Trauma Society (OTS) Open Fracture Classification System, developed in a setting with a high incidence of civilian gunshot fractures.

**Methods:**

From our prospectively collected database, we identified all patients with open tibia and femur fractures treated with intramedullary nailing over a 4 year period. All open fractures were retrospectively reclassified from the Gustilo-Anderson Classification system to the OTS Open Fracture Classification System.

**Results:**

One hundred and thirty-seven cases were identified. Ninety per cent of subjects were males. Their mean age was 34 years. The most common mechanism of injury was low-velocity civilian gunshot wounds (GSW) in 54.7% of cases. Soft tissue management was primary closure in 23.4% and soft tissue reconstruction in 24.1%. In 52.6% of cases (these all being secondary to civilian GSW), soft tissue management was healing via secondary intention. This is not included as a soft tissue management option in the OTS classification system. Fracture reclassification using the OTS Open Fracture Classification System was only possible in 47.5% of cases (Simple in 23.4%, Complex B in 24.1%).

**Conclusion:**

We conclude that the OTS Open Fracture Classification System is not inclusive of all open tibia and femur fractures as it does not cater for gunshot fractures. We propose a modification as follows: alter ‘wound debridement’ to ‘appropriate wound care’ and to subcategorise ‘Simple’ into type A and B: healing via secondary intention and primary closure, respectively.

## Introduction

The classification of open fractures is challenging. [1, 6, 12]. Numerous classification systems have been proposed, with numerous shortcomings [6]. Gunshot fractures, although typically categorised as open fractures, often exhibit incomplete inclusion within conventional open fracture classification systems. This necessitates their description in specialised gunshot-specific classification systems. Gunshot wound classification systems include the Red Cross Classification of War Wounds, the New Classification System of Gunshot Injuries in Civilians, Grading System for Gunshot Injuries to the Femoral Diaphysis in Civilians and the Intraarticular low-velocity gunshot injury Classification System. [15, 20, 25]. Civilian gunshot injuries are rising, with an increasing global burden. [2, 23, 24]. The literature on civilian gunshot classifications and clear management protocols is scarce [2, 10].

The Gustilo-Anderson (GA) Classification System remains the most widely used classification system for open fractures, with its use spanning over 45 years. However, it has modest interobserver reliability, with an overall *k* coefficient of 0.5. [5, 6, 22]. In 2020, the Orthopaedic Trauma Society (OTS) developed an open fracture classification system based on a prospective cohort study of two large clinical trials in the United Kingdom (UK) Major Trauma Network. [3]. This newer classification system objectively stratified open fractures after the index surgical debridement into two categories, simple (able to close primarily) and complex (subdivided into A, B and C, including bone shortening, soft tissue reconstruction and vascular repair respectively) [3], (Table 2). This correlated with patient-reported disability and quality of life in the first 12 months after the injury was sustained. Conversely, it did not correlate with deep surgical site infection 30 days after the injury was sustained. It is user-friendly and has unknown intra- and interobserver reliability for usage in the clinical setting. [1, 3]. It was therefore selected as the cornerstone for our study, to evaluate its inclusivity of our cohort.

An ideal open fracture classification system should include all open fractures, including those caused by a gunshot wound (GSW). We aim to classify all open tibia and femur fractures using the OTS Open Fracture Classification System in a setting with a high incidence of civilian gunshot fractures and propose a modified, more comprehensive version to include these injuries.

## Methods

Patients with open diaphyseal tibia and femur fractures were retrospectively identified from a prospectively collected cohort. This patient cohort was collected as part of the Human Immunodeficiency Virus in Orthopaedic Skeletal Trauma (HOST) study [21]. The data were extrapolated off an Excel database containing all patients aged 18 years and above who sustained open tibia and femur fractures, treated with intramedullary nailing between September 2017 and May 2021. Patients were excluded if they sustained closed fractures, had non-viable limbs, sustained open fractures that took more than 48 h to first surgical debridement, or were unable to follow-up for 12 months.

Open fractures in the database were classified using the GA classification by the primary operating surgeon after the initial surgical episode (98.4% being registrars in training), considering the soft tissue injury and radiological fracture morphology.

Both investigators, the principal investigator and Orthopaedic registrar (Z.L) as well as an Orthopaedic Trauma Surgeon (S.G) reviewed all extrapolated open fractures from the cohort and used relevant descriptive tools (soft tissue management, bony reconstruction and vascular repair) to reclassify them using the OTS Open Fracture Classification System.

All open fractures which were closed primarily from the cohort were classified OTS Simple. Fractures were treated with intramedullary nailing and had no bone shortening or deformation, therefore resulting in no Complex type A. Open fractures which were treated with soft tissue reconstruction, including split thickness skin grafts or flaps, were categorised into Complex type B. There were no patients with vascular injuries and, hence, no Complex type C. Open fractures which had no primary wound closure or soft tissue reconstruction but were treated definitively by healing via secondary intention were classified into another subgroup, as “unclassifiable”.

All open fractures now classified into the OTS Open Fracture Classification System had the following parameters evaluated; sex, age, number of tibia and femur open fractures, mechanism of injury, soft tissue management, and injury severity score (ISS).

## Results

**Fig. 1:**
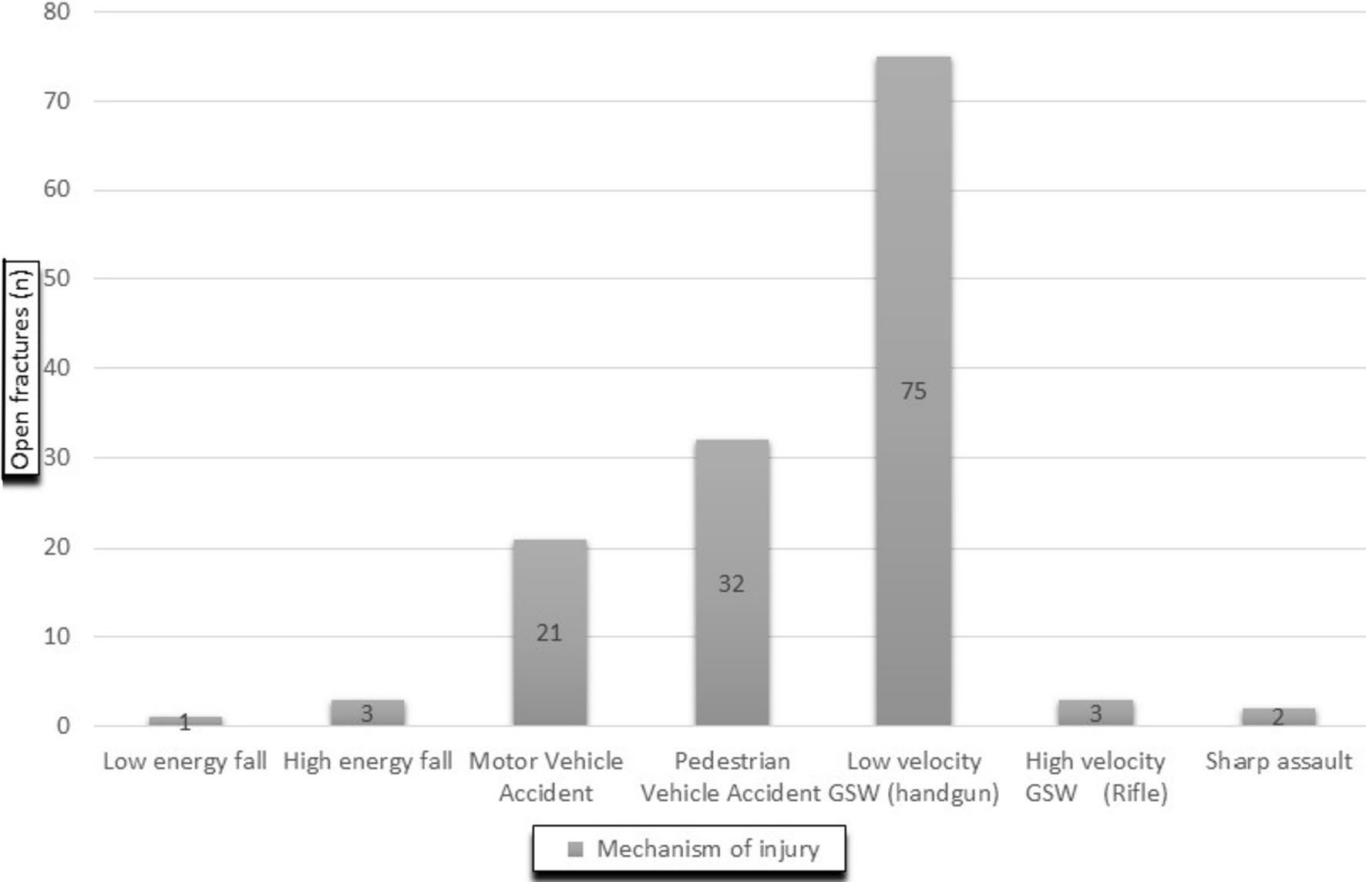
Correlation between mechanism of injury and incidence of open fractures

A total of 137 open tibia and femur fractures treated with intramedullary nails were included in this study. One hundred and sixty-one (161) open fractures were identified however twenty-four (24) were lost to follow-up and excluded. Males comprised 90% (*n* = 123) of the cohort and females 10% (*n* = 14). The mean age was 34 years, median age was 32.5 years, with an interquartile age range of 27–41 years. Patients with open fractures younger than 40 years of age were 84% of cases (*n* = 115). Tibia fractures made up 52.6% (*n* = 72) of the total long bone open fractures and open femur fractures made up 47.4% (*n* = 65) (Table 3). The mechanism of injury included low-velocity GSW (handguns) at 54.7% (*n* = 75) and pedestrian-vehicle accident (PVA) at 23.4% (*n* = 32). Both mechanisms emerged as the predominant contributors to the observed open fractures (Fig. 1). High-velocity GSW (rifle) counted for 2.2% (*n* = 3) of open fractures.

**Fig. 2:**
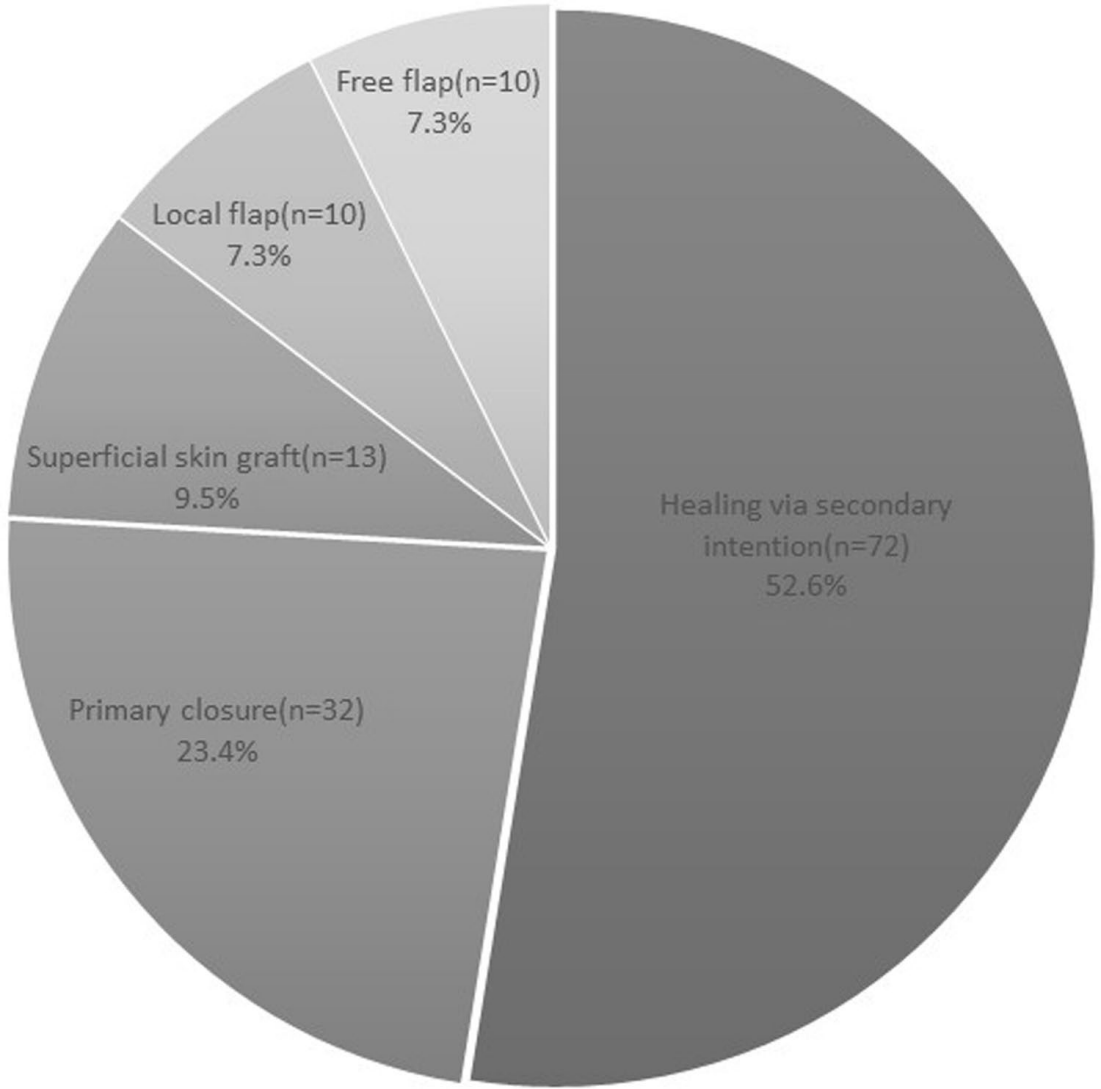
Percentage distribution of open fractures by soft tissue treatment modalities

Soft tissue cover was previously categorised under four headings, primary closure at 23.4% (*n* = 32), superficial skin graft at 9.5% (*n* = 13), local and free flaps making up 7.3% (*n* = 10) each. A fifth additional category was added in this study for those wounds managed by healing via secondary intention, with or without initial wound irrigation. This novel classification emerged as the predominant modality for soft tissue management among all instances of open fractures, representing 52.6% (*n* = 72) of the total cases (Fig. 2). The Injury Severity Score was 16 or less in 74.5% (*n* = 102) and 16 or more in 25.5% (*n* = 35).

The GA classification statistics were as follows: Type I with 67.9% (n = 93) with 79.6% (*n* = 74 of 93) being secondary to civilian GSW, Type II with 5.1% (*n* = 7), Type IIIA with 21.2% (*n* = 29) and Type IIIB with 5.8% (*n* = 8) with no Type IIIC open fractures. Using the OTS Classification System, the results were as follows: Simple with 23.4% (*n* = 32) and Complex type B with 24.1% (*n* = 33), with no Complex types A or C.

A total of 52.6% (*n* = 72) of open fractures defy classification according to the OTS Open Fracture Classification System, attributable to the absence of soft tissue intervention and reliance on healing through secondary intention (Table 1). Notably, these instances exclusively emanated from civilian gunshot wounds.

**Table 1:** A comparative analysis of open fracture classifications: the Gustilo-Anderson versus Orthopaedic Trauma Society Open Fracture Classification system

GA, *n* (%), host		OTS, *n* (%), orthopaedic registrar		OTS, n (%), orthopaedic consultant
I	93 (67.9)	Simple	32 (23.4)	32 (23.4)
II	7 (5.1)	Complex A	0	0
IIIA	29 (21.2)	Complex B	33 (24.1)	33 (24.1)
IIIB	8 (5.8)	Complex C	0	0
IIIC	0	Unclassifiable	72 (52.6)	72 (52.6)

## Discussion

Our study has demonstrated that the OTS Open Fracture Classification System is not inclusive of all open fractures, as it does not cater for low-velocity gunshot fractures. This mechanism of injury being the most prevalent in our cohort of open injuries. Our study demographics illustrate that most of the subjects were young adult males. In 2017, Martin et al. reviewed the cost of treating GSW in our clinical setting, with their cohort showing similar demographics [2].

Low-velocity GSW fractures ranked the highest cause of open long bone fractures at 54.7% (*n* = 75), followed by pedestrian-vehicle accidents at 23.4% (*n* = 32). Gunshot fractures are heterogenous open injuries, with each one behaving in a unique manner, needing thoughtful and tailored treatment strategies. [4]. Their treatment guidelines remain unclear due to no universally accepted GSW classification system. The Intraarticular Low-Velocity Gunshot Injuries Classification System has three categories: ultimate bullet location, contamination and fracture stability. Each category has three subdivisions. This aided in proposing a protocol specific to intraarticular gunshot injuries. [20]. The Red Cross Classification of War Wounds was described in 1992 by the International Committee of the Red Cross, based on war-related injuries and the characteristics of wounds sustained. [15]. The open fracture was assessed in theatre and scored according to the size of the entry and exit wounds, the presence of a cavity, a fracture, an injured vital structure and in the presence and absence of metallic bodies. Each category was assigned a letter and a numeral value. This makes this classification system convoluted and impractical for daily clinical use.

The New Classification System of Gunshot Injuries in Civilians, proposed in 2003, classifies gunshot injuries in civilians. It uses five divisions: energy dissipation, injured vital structures, the wound created, the severity of the bony injury, and the degree of contamination. It, too, was objective but too detailed and difficult to recall.

Long et al., in 2003, also looked at femur fractures secondary to gunshot injuries, affecting only the diaphyseal region, which they labelled as Zone 2 (Zone 1 being proximal to the lesser trochanter and Zone 3 being distal to the distal femoral metaphyseal/diaphyseal junction). They proposed a Grading System for Gunshot Injuries to the Femoral Diaphysis in Civilians classified into three grades depending on wound entry and exit sizes, muscle necrosis and radiological findings. [25]. This classification system is limited to only femoral diaphyseal GSW.

This lack of a standardised GSW classification system results in treatment based on the surgeon’s anecdotal experience. [15]. Management of soft tissue injuries in civilian gunshot injuries remains controversial due to most literature centred on research conducted on military firearms and missiles. [16]. A gunshot fracture with a simple, clean entry and/or exit wound and no exposed bone does not necessarily require formal surgical debridement. It is our local practice to allow these simple wounds to heal by secondary intention. [10].

In this study, we looked at low-velocity gunshot injuries only. Wound ballistics is the science of studying a penetrating projectile effect on the body. [17]. Civilian gunshot injuries are inflicted by low-velocity handguns, which generally result in less tissue damage than high-velocity weaponry such as rifles. [10, 15, 16]. Energy transfer also significantly contributes to the magnitude of injuries sustained from gunshots. [17, 18]. The ISS of less than 16 was 74.5%, almost three times more than the ISS of 16 or more at 25.5%. This was in keeping with the causation of low-velocity GSW, the top-ranked mechanism of injury. The higher percentage of patients with low ISS was due to our study population mostly represented by patients with isolated tibia and femur fractures (extremity injuries). These patients were physiologically stable. Patients with higher ISS were of a lower percentage due to fewer polytraumatised patients with other central injuries involving the chest, head and abdomen.

Using the OTS Open Fracture Classification System in this study, 32 open fractures were closed primarily at the first surgical episode. Thirty-three required soft tissue reconstruction: thirteen superficial skin grafts, ten local flaps and ten free flaps. The remaining 72 open fractures required no surgical soft tissue intervention, subsequently healing via secondary intention. These open fractures remained unclassifiable in this classification system. All 72 fractures were caused by low-velocity, civilian gunshots, in keeping with less severe soft tissue injury. The bony fractures in the study were all fixed with an intramedullary nail. This being our protocol for civilian gunshot fractures, as well as 1st generation Cephalosporin intravenous single dose, anti-tetanus toxoid and no entry/exit wound(s) debridement or surgical closure.

The GA Classification System is difficult to use in a setting of high gunshot fractures, as these injuries are diverse, with wound size not correlating with fracture morphology, level of contamination, soft tissue damage, periosteal stripping or vascular injury. [15]. Wound size is not a true reflection of associated tissue damage below the surface, energy dissipation and cavitation are still involved [15, 17, 19]. Numerous other open fracture classification systems exist, which were not selected as the foundation of this study.

Oestern and Tscherne, in 1984, also described a highly descriptive and poorly mutually exclusive classification system. This classification system categorises open fractures into four subgroups guided by the extent of soft tissue damage and muscle contusion. [7]. This, too, posed the same subjective pitfalls of the GA classification. [8]. There is also the Ganga Hospital Open Injury Score (GHOIS) which was proposed in 2004. It is a scoring system designed to aid decision-making of limb salvage or ablation of mangled limbs. It is not an open fracture classification system. [6, 9].

The Association of Study of internal fixation (AO-ASIF) in 2007 described an alphanumeric classification system, also looking at the intensity of skeletal, integumentary, musculotendinous and neurovascular injury in both closed and open fractures. It is extensive and structured, however not simplistic enough for daily use by clinicians. It omits the contamination category and is strictly based on the anatomical pathology of the affected limb. [6, 8].

In 2010, the Orthopaedic Trauma Association-Open Fracture Classification (OTA-OFC) was described as an objective open fracture classification system and an expansion of the Gustilo-Anderson classification, addressing its limitations. [1]. It uses five pathoanatomic characteristics and subdivides them into three categories of ascending severity, resulting in a final open fracture classification. The parameters included are skin, contamination, muscle, bone and arterial involvement. Although more comprehensive, the OTA-OFC is far too complex for day-to-day hospital use. [8, 11, 12].

## Limitations

The initial GA classification from the database was classified by a single primary surgeon. The reclassification was retrospective and based off the description of the open fracture injuries and not from photographs captured at the time post-operatively.

## Conclusion

We have shown that the OTS Open Fracture Classification System is not comprehensive, in that it does not include wound management for open fractures secondary to civilian GSW. To be scientifically sound, fracture classifications necessitate reliability, reproducibility, and clinical relevance. A proposed modification suggests the revision of ‘wound debridement’ to ‘appropriate wound care’ and the subdivision of the Simple category into A and B, signifying healing via secondary intention (A) and primary closure (B), respectively. Subsequent validation and reliability assessments are imperative and should be addressed in prospective studies.

## Figures and Tables

**Fig. 3:**
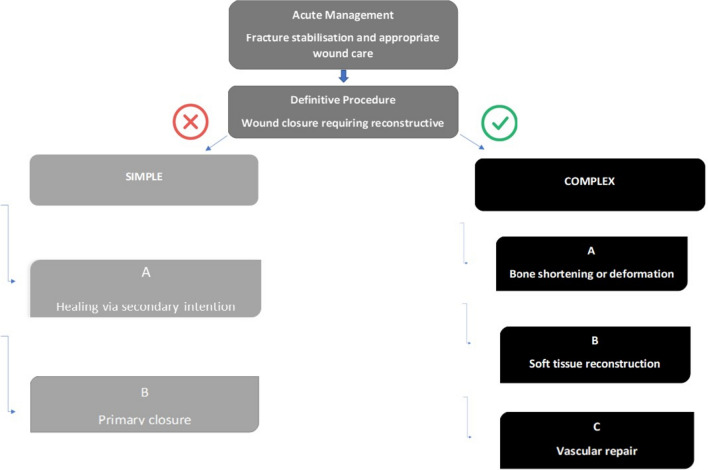
Modified Orthopaedic Trauma Society Open Fracture Classification system

## Data Availability

The data, along with all pertinent documentation, are securely archived within an Excel spreadsheet, and are accessible for reference as required.

## Appendix

See Fig. 3 and Tables 2 and 3.

**Table 2:** The Orthopaedic Trauma Society Open Fracture Classification system

Grading	Description
Simple	Primary closure of wound only, no reconstructive procedure
Complex A	Bone shortening or deformation
Complex B	Soft tissue reconstruction
Complex C	Vascular repair

**Table 3:** Demographic characteristics of the study cohort

Categories	
Sex, *n* (%)	
Male	123 (90)
Female	14 (10)
Mean age	34
Age, *n* (%)	
< 40	115 (84)
> / = 40	22 (16)
Total open fractures, *n* (%)	137
Tibia	72 (52.6))
Femur	65 (47.4)
Injury severity score, *n* (%)	
< / = 16	102 (74.5)
> 16	35 (25.5)
